# Actigraph Evaluation of Acupuncture for Treating Restless Legs Syndrome

**DOI:** 10.1155/2015/343201

**Published:** 2015-02-11

**Authors:** Weidong Pan, Mingzhe Wang, Mao Li, Qiudong Wang, Shin Kwak, Wenfei Jiang, Yoshiharu Yamamoto

**Affiliations:** ^1^Department of Neurology, Shuguang Hospital Affiliated to Shanghai University of TCM, 528, Zhangheng Road, Pu-Dong New Area, Shanghai 201203, China; ^2^Department of Neurology, Pudong New Area Hospital of Traditional Chinese Medicine, 460, Xiuchuan Road, Pu-Dong New Area, Shanghai 201200, China; ^3^Center for Disease Biology and Integrative Medicine, Graduate School of Medicine, The University of Tokyo, 7-3-1 Hongo, Bunkyo-ku, Tokyo 113-8655, Japan; ^4^Educational Physiology Laboratory, Graduate School of Education, The University of Tokyo, 7-3-1 Hongo, Bunkyo-ku, Tokyo 113-0033, Japan

## Abstract

We evaluated the effects of acupuncture in patients with restless legs syndrome (RLS) by actigraph recordings. Among the 38 patients with RLS enrolled, 31 (M = 12, F = 19; mean age, 47.2 ± 9.7 years old) completed the study. Patients were treated with either standard acupuncture (*n* = 15) or randomized acupuncture (*n* = 16) in a single-blind manner for 6 weeks. Changes in nocturnal activity (NA) and early sleep activity (ESA) between week 0 (baseline), week 2, week 4, and week 6 were assessed using leg actigraph recordings, the International Restless Legs Syndrome Rating Scale (IRLSRS), and Epworth Sleepiness Scale (ESS). Standard but not randomized acupuncture reduced the abnormal leg activity of NA and ESA significantly in week 2, week 4, and week 6 based on the changes in the clinical scores for IRLSRS and ESS in week 4 and week 6 compared with the baseline. No side effects were observed. The results indicate that standard acupuncture might improve the abnormal leg activity in RLS patients and thus is a potentially suitable integrative treatment for long-term use.

## 1. Background

Restless legs syndrome (RLS) is often associated with insomnia, specifically as difficulty with falling asleep, sleep maintenance, and sleep [1]. Thus, sleep disruption mainly comes from subjective reports from patients and from the clinical experience of physicians. Only a few studies focused on objective quantitative evaluations of RLS and they found an increase in sleep latency and a decrease in sleep efficiency only in patients with the most severe RLS symptoms [2–5]. A recent study demonstrated that sleep behavioral analysis by means of the patterns of actigraphic records, which is a small watch-type activity monitor equipped with a computer (*MicroMini-Motionlogger*, Ambulatory Monitoring, Inc, Ardsley, New York) [6], is helpful in understanding the mechanisms of subjective sleep perception in sleep disorders of Parkinson's disease [7]. Recently, acupuncture, an effective integrative therapy [8], has been shown to be a remarkably effective and well-tolerated agent for the treatment of serious neurological and psychiatric diseases [9–14]. Acupuncture is an ancient healing art and has been a significant component of Eastern Medicine for over 2,000 years [9, 15]. It has gained popularity in the United States as a modality of complementary and alternative medicine for certain disease entities and clinical conditions including neurodegenerative diseases. To our knowledge, there are no studies on actigraph use in RLS research or on the therapeutic effect of acupuncture in RLS patients. For these reasons, the aim of the present investigation was to analyze, in detail, the eventual baseline differences in actigraph patterns of patients with RLS and the eventual changes in sleep architecture and instability induced by the acute administration of acupuncture in patients with RLS.

## 2. Subjects and Methods

### 2.1. Subjects

A prospective single-blind sham-controlled study with consecutive enrolment of 38 subjects affected by idiopathic RLS was carried out on patients admitted to the Department of Neurology and Department of Acupuncture of Shuguang Hospital Affiliated to Shanghai University of Traditional Chinese Medicine (Table 1). The diagnosis of RLS was established according to the International RLS Study Group criteria [16]. In addition, for patients to be included in the study, they had to have had a mean frequency of symptoms during the last 6 months of more than twice per week, with a score of at least 20 on the International Restless Legs Syndrome Rating Scale (corresponding to severe RLS, IRLSRS) [17]. In order to evaluate the overall quality of sleep, outcome measures on quality of life were recorded using the Epworth Sleepiness Scale (ESS) [18]. Only patients who were between the ages of 35 and 85 years, were not taking any anti-RLS medication at the time of the study, and had never been treated for RLS (such as dopaminergic agents, benzodiazepines, opioids, and/or anticonvulsants) were included in the study. Furthermore, subjects suffering from known causes of secondary RLS (renal failure, anemia with iron deficiency, pregnancy, rheumatoid arthritis, recent anesthesia, fracture, or clinical myelopathy and peripheral neuropathy), other sleep disorders (e.g., narcolepsy, sleep terrors, sleepwalking, and sleep disordered breathing), or other movement disorders or who had any other medical conditions that would affect the assessment of RLS were excluded from the study.

All patients underwent a neurologic examination, routine blood tests (including serum iron and ferritin, B12 vitamin, and folate concentrations), electromyography (EMG), and electroneurography of the lower limbs. Patients with any abnormality in the above-mentioned tests or with an apnea-hypopnea index greater than 5 were also excluded.

### 2.2. Acupuncture Administration

**Figure 1:**
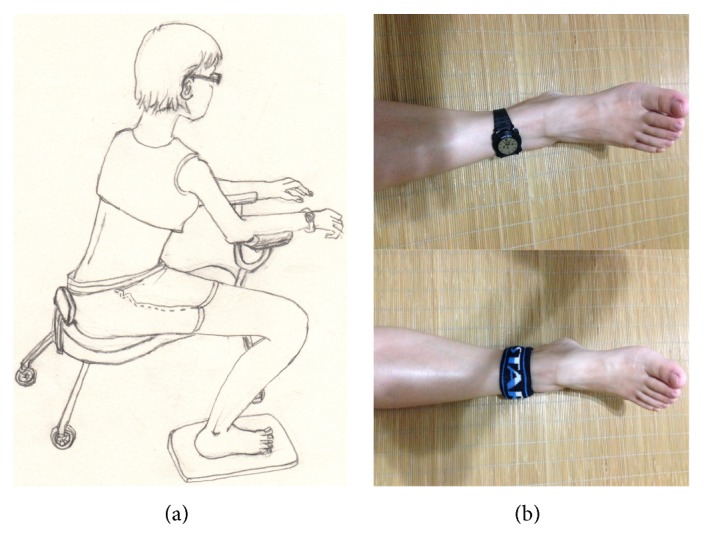
Patient position during acupuncture treatment (a) and location of actigraph equipment (b). The actigraph was worn on the ankle of the dominant leg (bilateral effected) or worse leg (top of (b)) and covered with a cuff (bottom of (b)) to avoid affecting sleep.

All subjects underwent 2 nocturnal actigraphic recordings [19] after an adaptation night and were randomly subdivided into 2 subgroups: a standard group (*n* = 19, traditional acupuncture stimulation) and a randomized (*n* = 19, randomized anatomic points) group. This allowed the acupuncturists to modify the acupuncture point prescription based on each patient's location of discomfort, with the intent of optimizing efficacy while facilitating reproducibility. All patients were treated around 16:00–18:00 pm each day while in a seated position (Figure 1(a)). A total of 12 acupoints (Shenshu (BL23, bilateral), Mingmen (DU4), Xuehai (Sp10, unilateral), Chenshan (BL57, unilateral), Taichun (LR3, bilateral), Zusanli (St36, unilateral), Sanyinjiao (Sp6, bilateral), and Taixi (Ki3, bilateral)) were used in patients in the standard group, while in the randomized group 12 randomized points that included sites distal to any zone of discomfort and avoided any acupoints local ashi tender points were selected. Acupuncture was performed by the same experienced acupuncturist. Huatuo needles 0.30 ∗ (25 mm–45 mm) (Suzhou Medical Supplies Co., Suzhou, China) were used. Puncture depth was 15 mm ± 2 mm for both groups. In order to develop needle sensation, after puncturing, the acupuncturist twirled the needle at an angle of 90–180° and frequency of 60–90 times/min and lifted and thrust at a range of 0.3–0.5 cm and frequency of 60–90 times/min. After manipulating the needle for 1 min, the needle was held in place for 30 min. During this 30 min, the physician repeated this manipulation for 1 min every 10 min [20]. All patients were treated for 6 weeks, 3 times per week. The study was approved by the Ethics Committee of Shuguang Hospital Affiliated to Shanghai University of TCM and was performed in accordance with the principles outlined in the Declaration of Helsinki. All patients gave their written consent for these procedures and were unaware of the content (standard or customized) of the treatment.

### 2.3. Nocturnal Actigraphic Recording

**Figure 2:**
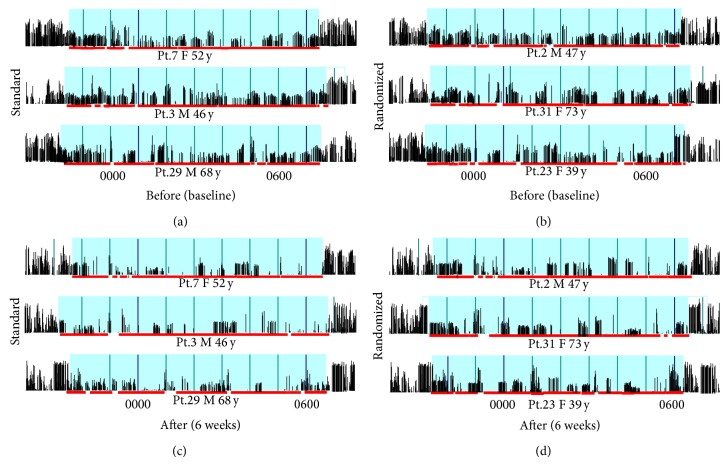
Qualitative changes in actigraph recording during sleep in 3 patients. (a) and (c) show the changes in actigraphic recordings of 3 patients with restless leg syndrome (RLS) before and after standard acupuncture treatment. (b) and (d) show the changes in actigraphic recordings in patients with RLS before and after randomized acupuncture treatment. The blue zone with a wide red line indicates period while patient was asleep.

Nocturnal actigraph recording was carried out after an adaptation night in a standard sound-attenuated (noise level to a maximum of 30 dB normal hearing level) sleep room (wards associated with the Department of Acupuncture or Department of Neurology, Shuguang Hospital Affiliated to Shanghai University of TCM, or at home). Subjects were not allowed to consume caffeinated beverages from the afternoon preceding the recordings and were allowed to sleep until spontaneous awakening in the morning. Lights-out time was based on individual habitual bedtime and ranged between 22:30 and 23:00. All subjects wore an actigraph on the ankle of the affected dominant side or worse side (Figure 1(b)) for 2 consecutive days in the series time windows (2 weeks each) during the 6-week observation period. We asked the patients or the family members to write down the diary and we also used the light detecting device (inserted in the actigraph) to distinguish the series time periods in order to avoid the miss movement or abnormal activity. Each subject had to be tested 4 times: before treatment (baseline) and 2 weeks, 4 weeks, and 6 weeks after starting the treatment. Zero-crossing counts were recorded every minute by the actigraph to register and quantify the level of physical activity [21, 22]. After recording, the data were transmitted to an external computer by software installed on the device. We plotted the activity scores for two consecutive days to determine the nocturnal activity of each patient (Figure 2). The data acquired from the actigraph during the observation period were separated into two time periods for analysis: early sleep activity (ESA, each first hour of night bedtime activity) and the nocturnal activity (NA, all nocturnal bedtime activity).

The primary outcome was the change in activity level of NA and ESA of the legs of the RLS patients. The second outcome was the change in IRLSRS and ESS scores. The outcome measures were assessed at baseline and every 2 weeks during the 6-week intervention.

### 2.4. Statistical Analysis

Repeated-measure ANOVA was conducted to test the differences among changes in outcomes at baseline and every 2 weeks for 6 weeks. When a significant difference was detected, a post hoc test (Bonferroni test) was conducted between the standard and randomized groups in order to compare the levels of NA and ESA, and the IRLSRS and ESS scores. Differences at baseline between the standard group and randomized group were analyzed using the *t*-test. A significant difference was defined as *P* < 0.05. SPSS Windows version 17.0 was used for statistical analyses. All data are expressed as the mean ± standard deviation.

## 3. Results

Seven patients dropped out of the study: two in the standard group and two in the randomized group were unable to tolerate the pain of acupuncture, while one in the standard group and one in the randomized group dropped out due to a conflict with other physical treatments (one was massage and one was transcranial magnetic stimulation) prescribed for concomitant diseases. One patient in the standard group dropped out due to the poor response of the treatment. Neither physical examination nor laboratory tests revealed any adverse changes after additional treatment in either group at the end of the study.

At the end of the investigation, 15 patients in the standard group and 16 patients in the customized group were included for statistical evaluation. The post hoc test revealed no significant differences in age, sex, age of RLS onset, duration of RLS diagnosis, baseline of activity level, or clinical severity between the standard group and randomized group (Table 1).

Figure 2 presents the qualitative changes in the actigraph recording during sleep before and after treatment. Patients in both the standard and randomized groups showed decreased actigraph patterns after 6 weeks of acupuncture treatment (Figures 2(c) and 2(d)) compared to before treatment (Figures 2(a) and 2(b)), although those in the standard group seemed to have more decreased patterns (Figures 2(a) and 2(c)) than patients in the randomized group (Figures 2(b) and 2(d)).

**Figure 3:**
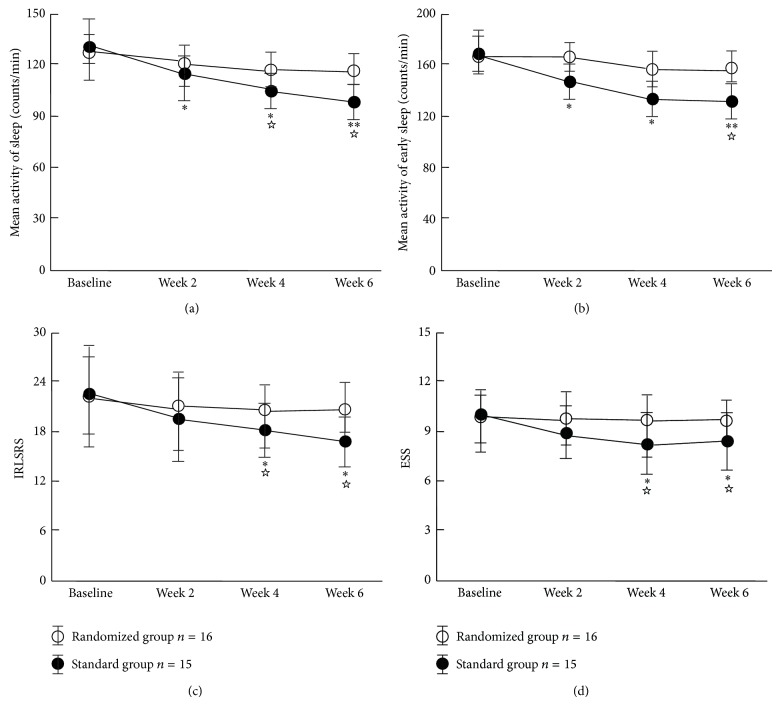
Quantitative changes in actigraph recordings and clinical evaluation scores. (a) shows the changes in sleep time mean activity scores, (b) the changes in early sleep time mean activity scores, (c) the changes in IRLSRS scores, and (d) the changes in EES scores every 2 weeks after 12 weeks of treatment by standard or randomized acupuncture. Solid circles indicate standard acupuncture treatment and hollow circles randomized acupuncture treatment; ^*^ *P* < 0.05 and ^**^ *P* < 0.01 indicate the changes in standard treatment compared with baseline, while *P* < 0.05 indicates the changes in standard treatment compared with randomized treatment. IRLSRS: International Restless Legs Syndrome Rating Scale; ESS: Epworth Sleepiness Scale.

Compared with baseline, the standard group showed significantly decreased patterns from week 2, week 4, and week 6 when evaluated in terms of the effects by NA and ESA in leg actigraph recordings (Figures 3(a) and 3(b)), while significant improvements were observed from week 4 and week 6 based on the IRLSRS and ESS scores (Figures 3(c) and 3(d)). Significant differences were also found from week 4 and week 6 for NA, week 6 for ESA, week 4 and week 6 for the IRLSRS score, and week 4 and week 6 for the ESS score between the standard group and randomized group. No significant improvements in these parameters were observed between before and after acupuncture treatment in the randomized group (Figures 3(a)–3(d)).

## 4. Discussion

Based on the clinical properties of RLS, an increase in abnormal leg activity should be one objective parameter that represents an uncomfortable symptom in these patients [5]. Our RLS study is the first investigation to directly compare standard and customized acupuncture treatments in RLS. The decision to use the NA and ESA scores as the primary endpoint reflects the standard approach at the time the study was planned. In addition, an actigraph provides an objective assessment of leg activity. The IRLSRS and ESS scores have often been used in evaluating RLS in previous studies [5, 23]; however, they are subjective assessments so we used them as secondary endpoints in the current study.

Our study results suggest that standard acupuncture is comparably effective in the treatment of RLS patients. Significantly greater reductions in the leg activity scores of NA and ESA were found with standard acupuncture when compared to randomized acupuncture (Figures 2(a) and 2(b)). Furthermore, the improvements observed in IRLSRS and ESS scores reflected moderate disease characteristics as a consequence of changes at week 4 and week 6 in the standard acupuncture group (Figures 2(c) and 2(d)).

Acupuncture is an integral part of traditional Chinese medicine (TCM), which dates back more than 2000 years. In recent years, the use of acupuncture as an adjuvant treatment to the management of various obstetrical and gynecological conditions has gained increasing popularity worldwide [24]. It also has been studied in the treatment of serious neurological diseases such as neurodegeneration diseases [25, 26]. As one preclinical symptom of Parkinson's disease, RLS has been proposed to be one of the initial stages of neurodegenerative disease [27]. In the theory of TCM, RLS is considered a deficiency of “Yin” and “Xue” (blood) of the legs, because “Yin” and “Xue” are in effect at night and function to relax the mind and body. According to TCM, the activity and function of legs are controlled by liver function, and, thus, the deficiency of “Yin” and “Xue” of the liver at night is the main cause of RLS. Shenshu (BL23, bilateral), Mingmen (DU4), and Chenshan (BL57, unilateral) have effects on uncomfortable waist and leg symptoms, Shenshu (BL23, bilateral) and Mingmen (DU4) can also improve “Shen Qi” (energy and immunity) and increase the “Yin” of the waist and legs, and Xuehai (Sp10, unilateral) increases “Xue” (blood) of the body, which means it can tranquilize the legs. Taichun (LR3, bilateral), Zusanli (St36, unilateral), Sanyinjiao (Sp6, bilateral), and Taixi (Ki3, bilateral) increase both “Yin” and “Xue” (blood) of the body. If these acupoints are treated properly, the legs should be quiet at night. According to the Huang Di Nei Jing Su Wen, acupoint specificity is an essential principle [28] which means that the therapeutic efficacy results mainly from the correct selection of acupoints and the effects of acupuncture are completely related to appropriate acupoint selection during the treatment [29].

Guo et al. [30] demonstrated that acupuncture at the Jianshi-Neiguan acupoints (P5-P6, underlying the median nerve) inhibits central sympathetic outflow and attenuates excitatory cardiovascular reflexes through an opioid mechanism, suggests acupuncture, might regulate autonomic function. Zhou et al. [31] found nociceptin in the spinal cord mediates part of the acupuncture-related modulation of visceral reflex responses. Lin and Chen [32] found that the autonomic nervous system (ANS) seems to indicate its connection with acupuncture. The inflammatory reflex (via the ANS) might be a crucial part of antihyperalgesia elicited by acupuncture, and this reflex, which regulates the immune system in the organism, can elucidate not only the mechanism of acupuncture analgesia but also the mechanism of acupuncture applied to other inflammatory conditions. Innovation of functional image study enables us to analyze the responses of cortex on living human body to acupuncture. It might interpret the mechanism on endogenous opiates and on the autonomic nervous system, in modifying the mechanisms of RLS and its sensorimotor manifestations.

All patients were treated while in the sitting position (Figure 1(a)), and this is one of our treatment strategies. While acupuncturists in China, Korea, and Japan generally insert the needle at the most convenient site, defined and unified positions have not been identified, although acupoints have been standardized. This may have an influence on the outcome of the acupuncture. If a site is punctured with differing angles, there will be errors in the locations of the acupoints, which may cause errors. In the present study, we were the first to define unified puncture positions, which is in contrast with previous acupuncture studies that did not.

Overall, treatment with standard acupuncture and randomized acupuncture was well tolerated. During the administration of acupuncture treatments, no dizziness, nausea, severe pain, or skin eruption occurred. The side effects were much less than those seen with the dopamine agonist pramipexole or levodopa/benserazide in other studies [33–35].

Although the two clinical scores indicate an improvement with standard acupuncture after week 4 and week 6, these effects were observed after each nocturnal duration so the scores might be subjective because they depended on awake conditions, such as dream, insomnia, and some somatization disorders.

The clinical RLS scores and actigraph patterns showed the same trends when evaluating the effects of RLS treatment. The small number of subjects (total 31 patients with RLS) and shorter evaluation duration (only 6 weeks) is one limitation of our research. Another is the lack of evaluation of the correlation between clinical scores and actigraph patterns. In the future, we plan to use more patients with RLS and design a more integrative study.

Six weeks of standard acupuncture treatment is effective for patients with RLS. The actigraph monitoring used in this study seems to be a more objective and direct tool for evaluating the effects. Its small size, good quality, and ability to be covered by a cuff may enable the recording of the true activity levels of patients and eventually may be suitable for quantitative clinical RLS evaluation.

## Figures and Tables

**Table 1:** Basal characteristics of all patients with restless leg syndrome.

	Standard (*n* = 15)	Randomized (*n* = 16)
Male/female	6/9	6/10
Age (y)	47.3 ± 9.6	46.8 ± 11.3
Age of RLS onset (y)	44.5 ± 6.1	43.7 ± 7.3
Duration of RLS diagnosis (y)	3.1 ± 2.3	2.8 ± 2.7
Baseline of nocturnal activity	132.9 ± 36.5	130.3 ± 38.7
Baseline of early sleep activity	166.8 ± 37.1	163.2 ± 39.4
Baseline of IRLSRS	22.3 ± 6.9	22.1 ± 7.2
Baseline of ESS	9.9 ± 4.1	9.6 ± 5.2

Note: RLS: restless leg syndrome; IRLSRS: International Restless Legs Syndrome Rating Scale; ESS: Epworth Sleepiness Scale.
